# In-Plane Shear Strength of Single-Lap Co-Cured Joints of Self-Reinforced Polyethylene Composites

**DOI:** 10.3390/ma14061517

**Published:** 2021-03-19

**Authors:** Felipe Vannucchi de Camargo, Eduardo da Silva Fernandes, Carla Schwengber ten Caten, Annelise Kopp Alves, Carlos Pérez Bergmann, Giangiacomo Minak

**Affiliations:** 1 Post-Graduation Program in Mining, Metallurgical and Materials Engineering, Federal University of Rio Grande do Sul, Av. Osvaldo Aranha 99, Porto Alegre 90035-190, Brazil; annelise.alves@ufrgs.br (A.K.A.); bergmann@ufrgs.br (C.P.B.); 2 Department of Industrial Engineering (DIN), Alma Mater Studiorum—Università di Bologna, Via Fontanelle 40, 47121 Forlì, Italy; giangiacomo.minak@unibo.it; 3 Post-Graduation Program in Industrial Engineering, Federal University of Rio Grande do Sul, Av. Osvaldo Aranha 99, Porto Alegre 90035-190, Brazil; eduardo.fernandes@ufrgs.br (E.d.S.F.); carla.caten@ufrgs.br (C.S.t.C.)

**Keywords:** stress analysis, mechanical properties, thermoplastic composites, design of experiment, UHMWPE, LDPE

## Abstract

The present study introduces the analysis of single-lap co-cured joints of thermoplastic self-reinforced composites made with reprocessed low-density polyethylene (LDPE) and reinforced by ultra-high-molecular-weight polyethylene (UHMWPE) fibers, along with a micromechanical analysis of its constituents. A set of optimal processing conditions for manufacturing these joints by hot-press is proposed through a design of experiment using the response surface method to maximize their in-plane shear strength by carrying tensile tests on co-cured tapes. Optimal processing conditions were found at 1 bar, 115 °C, and 300 s, yielding joints with 6.88 MPa of shear strength. The shear failure is generally preceded by multiple debonding-induced longitudinal cracks both inside and outside the joint due to accumulated transversal stress. This composite demonstrated to be an interesting structural material to be more widely applied in industry, possessing extremely elevated specific mechanical properties, progressive damage of co-cured joints (thus avoiding unannounced catastrophic failures) and ultimate recyclability.

## 1. Introduction

Composite materials are widely known for being able to offer enhanced mechanical properties as a result of the interaction of their constituent materials, which would perform less efficiently if considered solely. This concept may be applied involving metallic, ceramic or polymeric matrices, and it generally makes use of fibrous reinforcement due to its advantageous slender shape for manufacture-inherent defects with reduced size, along with the possibility of orientation, which allows cost- and weight-effective designs.

### 1.1. Thermoplastic Composites and Recycling

Polymeric composites may have thermoplastic or thermoset matrices which differ in many ways, from their mechanical properties to manufacturing processes. The ability to go through reprocessing, often with barely no resulting degradation, is among the most interesting features of thermoplastics, which have been widely studied by many authors, mainly for polypropylene (PP) [1,2,3] and polyethylene (PE) [4,5,6,7,8,9,10], which are the main polymers used in the automotive sector and in industry in general, respectively.

PE is a very versatile polymer with a diversified grade range depending on its molecular structure. Several previous researches approached its reprocessability in the cases of high-density (HDPE) [4,5,7,9,10] and low-density polyethylene (LDPE) [7,8,10]. PEs with low molecular mass and high degree of branching, such as LDPE, have a very low sensitivity to degradation [7,8], even if minorly affected by the predominantly deterioration mechanisms of chain branching and crosslinking [10]. This degradation is especially neglectable if antioxidants (e.g., phenolic, phosphite) are added during reprocessing, which not only preserve the melting flow index of the material, but may also increase the elongation at break of the polymer even after five cycles, thus solving a classic issue of PE reprocessing [10]. In both HDPE and LDPE cases, the property decrease is far from drastic, once they do not decay up to the second cycle and can even stabilize after the seventh or eighth [7], demonstrating that well-controlled recycling procedures of PE lead to only minor property losses.

Unlike thermoplastic polymers that can be remelted, thermosets cannot be due to their crosslinked nature [11,12]. In this case, it becomes cheaper to dispose these materials in landfills or to degrade them with high-temperature or toxic chemicals [11], whereas none of the aforementioned alternatives are environmentally friendly. Thus, given that thermosets such as epoxy and polyester constitute the biggest resin market share in the composite industry, the difficulty in creating a sustainable lifecycle for these products becomes a matter of concern, which encourages the usage of thermoplastics, such as PE, on a larger scale.

### 1.2. Polyethylene

PE is a thermoplastic, flexible, lightweight, translucent and water-aging-resistant [13] polyolefin (unlike most regularly used thermoset composite matrices [14]), representing the most widely applied class of polymers in volume worldwide [15,16]. It can be polymerized via free radical, resulting in a branched structure (LDPE), or ionically, resulting in low-branched linear carbon chains, which attribute a high degree of crystallinity to the material (HDPE) [17]. A higher molecular weight leads to a higher tensile strength and thermal stability due to the increase in molecular entanglement among crystallites [18]. PEs with molecular weights higher than 1 × 10^6^ Da enter the class of ultra-high-molecular-weight PE (UHMWPE), produced via Ziegler–Natta polymerization with highly oriented crystalline lamellae [19], known for good chemical resistance and mechanical performance, in some cases even outperforming carbon fibers in terms of specific strength.

The unique strength-to-weight ratio and cut resistance properties of these fibers enable them to be successfully adopted in several critical industrial applications such as biomedical devices [20], body armor, composite helmets, offshore mooring lines [21], vehicle suspensions [22], protective gloves, parasails and parachutes [17]. Nevertheless, the potential applications of PE are strictly related to their temperature, as the melting temperature (T_m_) of PE does not suit it to high-temperature usage.

UHMWPE is especially interesting for impact and ballistic applications [23,24] due to its high specific toughness, high modulus and low density, which are all great attributes for energy dissipation. The in-plane shear properties of UHMWPE composites have been studied by a few authors. Cline et al. [25], from the United States army, demonstrated that protective helmets reinforced with UHMWPE instead of polyaramid become lighter and more resistant, underlining the importance of studies such as the present one on the effects of temperature, pressure, and time on hot-pressing PE composites given its high sensitivity to the effects of processing.

### 1.3. Self-Reinforced Polyethylene Composites

Self-reinforced composites (SRCs) (also known as single-polymer, single-phase, homogeneous or mono-material [26]) are composites where the matrix and reinforcement belong to the same polymer family, but with distinct mechanical and thermal properties. The first study concerning SRCs was published back in 1975 by Capiati et al. [27], who analyzed oriented PE filaments in a lower-melting-point PE matrix. However, SRCs are not restricted to polyolefins (PE, PP); they also include polyesters (polylactic acid—PLA, polyethylene terephthalate—PET, poly methyl methacrylate—PMMA) and natural polymers (protein, cellulose and starch-based) [28]. The processing temperature window of such composites is selected between the T_m_ of the matrix and the T_m_ of the reinforcement, whereby the first is lower than the latter, to allow molding without affecting the properties of the reinforcement. The main mechanical advantage of SRCs relies on the improved fiber–matrix adhesion favored by molecular entanglements, providing enhanced stress transfer via the interphase improving properties directly related to the interfacial bond such as tensile and compressive strength, work-to-fracture, creep and fatigue [27]. This is topped by their low density, whereas other fibers such as carbon, aramid, and glass are denser [26].

Furthermore, the most appealing advantage of SRCs involves their sustainability. After all, given the technological barriers to efficiently recycle thermoset composites, SRCs disregard the concern for innovative recycling technologies, as the material itself does not require complex processes beyond remelting its components altogether. Karger-Kocsis et al. [26] underlined that SRCs are strictly connected to the need for developing low-density composites with ultimate recyclability. Gao et al. [28] stated that SRCs constitute a class of polymeric composites with high value as a recyclable product due to their homogeneity, which is particularly interesting given that chemical additives are not needed to enhance the mechanical synergy of the constituents that are made from the same base-material, thus enhancing even more biodegradability. For their good properties, among SRCs, most works in the literature investigated either PP [1,29,30,31] or PE [32,33,34,35,36,37,38,39,40,41,42,43,44].

A higher modulus of reinforcement leads to better mechanical behavior of the composite, and a higher molecular weight leads to a wider temperature processing window. Thus, naturally, several works evaluated the usage of UHMWPE as reinforcement in either LDPE or HDPE matrices, which was shown to improve the tensile strength, elastic modulus, and creep resistance of PE SRCs [35]. Arazi et al. [34] demonstrated the elevated ballistic properties of UHMWPE/HDPE SRCs, where the viscoelastic damping of this composite contributed to energy absorption. Hees et al. [40] demonstrated that PE SRCs with UHMWPE nanophases formed during processing were able to increase the wear resistance, toughness, stiffness, and strength of this class of composite. Poulikidou et al. [44] presented a case study in which a PE SRC was used for the production of truck exterior panels, resulting in 25% lower environmental impact than the previous solution with glass fiber-reinforced composites.

One of the most critical aspects for the performance of PE SRCs is processing, since temperature, cooling rate, pressure, and time may be crucial to determine the resulting mechanical behavior. These parameters may influence the interaction between constituents by avoiding overheating-related structural impairment, and by controlling the degree and shape of crystallization in the interphase. In PEs, the melting temperature is approximately 110 °C for LDPE, 130 °C for HDPE, and 135 °C for UHMWPE [26]. Aligned with the particularities of manufacturing, the effects of processing parameters are also crucial when bonding two tapes, a common situation in industry. After all, beyond the general use of tapes for laminates, PE SRCs are highlighted for their possibility to be co-cured on the surface of PE structures, thereby creating local reinforcements by melting the matrix with the substrate; and for their possibility to be used as belt-like reinforcements by co-curing both ends of the tape in an adhesive-free joint. Potential applications include bearings, drums, pallet boxes, and rotomolded containers in general.

### 1.4. Lap-Bonded Joints

Structures formed by the assembly of two or more parts are commonly used to overcome the impracticality of manufacturing large structures in a single piece due to processing and cost limitations [45]. The resulting joints are responsible for ensuring effective load transfer between parts, thus maintaining the integrity of the linked components [46]. For composites, there are two main joining methods: mechanical fastening (which infers undesired and prejudicial stress concentration spots and an overall weight increase caused by additional fasteners [47]) and adhesive bonding (which provides load transfer with more uniform distribution over larger areas) [48]. Adhesive bonding may be performed by secondary bonding, involving the usage of adhesives between the parts [49,50], or by co-curing, where the matrix of the composite is cured in contact with the desired adherend and, hence, cure and bonding happen simultaneously. For thermosets, the co-cure has to be performed during the cure of the second adherend, be it another composite [45,51,52,53,54,55] or a metal [56,57,58,59]. Kim et al. [51] evidenced the superior failure strength of co-cured joints without adhesive for carbon/epoxy laminates compared to those with adhesive or secondary bonding. These joints are also advantageous when the composite is subjected to thermal loads, as the joint and adherends possess the same thermal conductivity and expansion coefficients, unlike when adhesives made from a second (or third) material are involved [60].

The proper design of composite co-cured joints is a challenging field of study [60] which has been approached by several authors, where specific parameters (temperature, pressure, and time) must be perfected for each kind of bonded material to achieve improved adhesion, without impairing the polymer properties [61], and to allow reliable failure prediction via specific mechanisms. As joints are regions with naturally increased stress concentration, they should be designed to minimize peel and cleavage stresses and improve shear and compressive resistances. The joint types analyzed in the literature include single-lap, double-lap, scarf, and stepped-lap, with the first case being the most common due to its simple and efficient layout [60].

The advantages of thermoplastics, thus, facilitate such joining technique. Specifically, their inherently lower melting temperatures and possibility to be remelted allow much simpler temperature-induced bonding procedures, dismissing long thermal cycles under controlled atmospheres. Nevertheless, studies on co-curing processes for bonding two thermoplastic composite adherends are fairly scarce. One of the most representative studies on the optimization of co-cured thermoplastic bond processes is perhaps the study carried out by Hussein et al. [56], which determined the optimal pressure, temperature, and time to bond PE plates to an aluminum alloy using hot-press. Although interesting conclusions were drawn, since their research adopted a polymer–metal interaction, the conclusions are mostly not applicable to a polymer–polymer case. For instance, pressure was the most effective processing parameter to increase the shear strength of the lap joint, reaching its maximum at 10 bar; however, this trend would hardly apply when joining fiber-reinforced thermoplastic tapes, as the fibers would be damaged and their orientation would be shifted.

Despite the high industrial and environmental potential of co-cured thermoplastic joints, particularly in the case of high-performance fiber-reinforced composites, there is no general agreement on failure prediction methods (since the failure mode varies depending on the processing parameters [60]) or an appropriate technical standard that covers the mechanical assessment of such joints. ASTM D5868 [62], D3163 [63], D4896 [64], and D5573 [65] are the closest standards, but they all regard adhesively bonded joints, which generally perform much differently than co-cured ones. ASTM D3163 [63] specifically states that the method is not intended for use on anisotropic adherends such as reinforced plastics. Furthermore, processing parameters for bonding such as temperature are variable as a function of the prescribed conditions determined by the adhesive manufacturer [62], whereas additional pressure [62] and even joint overlap lengths [63] are optional.

These factors encourage studies such as the present one on joint processing optimization, especially for UHMWPE composite tapes that impose uniquely challenging mechanical assessment routines due to their inherently low friction coefficient, chemical inertness, and absence of polarity [24,66].

### 1.5. The Present Work

Supported by the literature review above, this work aims at targeting the assessment of single-lap shear strength of co-cured joints in all-PE composite tapes, determining the ideal bond processing conditions (temperature, pressure, and time) for a material that is highly sensitive to the effects of processing [25]. The optimal mechanical resistance was achieved using the design of experiment (DoE) method of response surface modeling (RSM), as recommended by Montgomery [67]. This research subject becomes especially appealing considering the high-performance nature of the tape analyzed, which is reinforced by UHMWPE (10 time stronger than steel, lighter than water, and highly recommended for extreme applications such as ballistics [68]) and embedded in a recylcled LDPE matrix. This research underlines the environmental concern related to PE, the most consumed general-purpose polymer [7] and, consequently, the main source of plastic waste in the world [5], by assessing a strong and stiff SRC PE tape [33] with ultimate recyclability.

The processing window gap was identified via differential scanning calorimetry (DSC) of the components, which were individually characterized by Fourier-transform infrared spectroscopy (FTIR). Tensile tests were performed in the fiber, in the matrix, and in the composite for comparison with the shear resistance of the single-lap co-cured joint. With the aim of overcoming the lack of reliable models for structural load-bearing applications for this kind of joint and material [60], the authors present a regression equation to estimate the resistance of the joint and analyze the predominant fracture mechanisms using light microscopy (LM).

## 2. Materials and Methods

### 2.1. Material Characterization

Both constituents of the composite studied herein, i.e., the reprocessed LDPE matrix and reinforcing UHMWPE fibers, were characterized by FTIR with a Shimadzu IRAffinity-1 (Kyoto, Japan) and by DSC with a Netzsch 404 F1 Pegasus (Selb, Germany), with the aim of determining their T_m_ and the consequent temperature window available for optimization of co-cured joint processing.

To establish a baseline, literature values for fiber and matrix properties are listed in Table 1, in which density was provided directly by the material supplier. Nevertheless, knowing that experimental studies may present variations in properties, the tensile mechanical resistance of the individual constituents and the composite considered herein were assessed experimentally.

**Figure 1:**
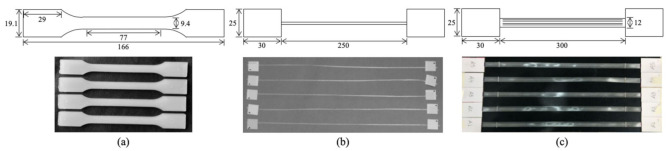
(**a**) LDPE, (**b**) UHMWPE, and (**c**) composite tape specimens in detail. Dimensions in millimeters (not to scale).

For the matrix, the technical standard ASTM D638 [69] was adopted and applied to four 3.65 mm thick injection-molded dogbone-shaped specimens similar to type I. The dimensions of the specimens are shown in Figure 1a. The average and standard deviation measurements of their dimensions were dismissed, as indicated by this ASTM standard, due to the considered manufacturing method that is able to produce identical specimens. The test was carried out using an MTS 810 universal testing machine (Eden Prairie, MN, USA) at 500 mm/min.

To overcome the inherent difficulties to test UHMWPE fibers due to their very low friction coefficient, a specially designed fixture was considered [75]. The yarn specimen (Figure 1b) preparation was reproduced according to the method of Camargo et al. [21], which consists of ends with thin tabs bonded with cyanoacrilate, and a total length of 310 mm in which each grip holds a 30 mm long tab and the distance between them is 250 mm. The test was carried out according to ASTM D885 at 250 mm/min, i.e., the absolute value of the nominal gage length [76], in a Remet (Ceretolo, Italy) TC10 with screw-tightening grips. There was no slippage during the tests. A preload of 10 N was adopted.

For the composite, testing single PE tapes instead of stacked laminae is advised to avoid the combination of high stiffness and strength with low interlaminar shear strength, which infers loading to the whole cross-section, thus yielding misleading results, for instance, in the case of dogbone-shaped specimens [24]. To overcome this obstacle, Zhou et al. [77] focused on creating a more efficient test apparatus on the basis of an expandable toroid that subjects ring-shaped yarns and tapes to tension, thereby obtaining successful results. Similarly, Heisserer et al. [75] considered a roller grip to test the tapes. Although valid, these testing procedures might not be feasible in most laboratories worldwide that do not possess such particular fixtures. On the other hand, the simplistic specimen preparation described by Camargo et al. [21], accounting for cyanoacrylate-sandwiched terminations for UHMWPE yarns, was applied in the present work and found to be highly effective.

As informed by the manufacturer, the composite had 65.6% fiber volume, and it was 0.092 mm thick and 12 mm wide. To verify these specifications, linear measurements were carried out on five specimens with 1 m on a Kern analytical scale with 1 × 10^−4^ g resolution. For tensile tests, five specimens were prepared using the same method adopted for yarns, except with 300 mm of gauge length, resulting in no slippage during testing (Figure 1c). They were tested on the same test machine used for the yarns. A test velocity of 100 mm/min was considered after a 10 N preload was applied through 50 mm/min. The tape tests were designed for a single composite layer to avoid slippage due to the influence of interlaminar stress transfer issues of this particular material [23], as explained above. The test routine described is in agreement with the standard ASTM D882 [78] for thin plastic sheets, which also covers anisotropic materials. The only divergences are the gage length of 300 mm that was adopted instead of 250 mm, to allow more conservative tests and to enhance the minimization of any potential grip slippage effect, and the velocity of 100 mm/min with a strain rate of 0.3 min^−1^ instead of 30 mm/min and 0.1 min^−1^ to enable comparisons with tests of the same material but using roller grips carried out by the tape manufacturer, thus double-checking the feasibility of using tabs bonded with cyanoacrylate as terminations.

### 2.2. Single-Lap Joint Shear Tests

Considering that joints represent one of the most challenging design features to tackle in composites due to their discontinuity and high local stress concentration [60], the single-lap joint shear tests were conducted on the basis of an optimization study for the bonding parameters of pressure, temperature, and time of processing by hot press. The value range defined for each parameter was selected not only according to similar studies [45,51,56,58,59,79,80], but also according to observations of the composite in terms of its thermal properties and preliminary co-cure assessments. The specimens were identical with regard to their overall dimensions to those subjected to tensile tests. The minimum length of 25.4 mm of each termination inside each test grip, as advised by the similar-purpose standard ASTM D5868 [62], was fulfilled for the proposed specimen geometry. Ink marks were made close to each termination to check for slippage, which did not occur in any of the tests carried out. The only difference in specimen geometry was that a thicker tape of 0.288 mm thickness was used with the aim of submitting a slightly more robust composite to the parameter processing study, which, in order to be statistically significant, needed to achieve the extremes of the temperature–pressure window available.

**Figure 2:**
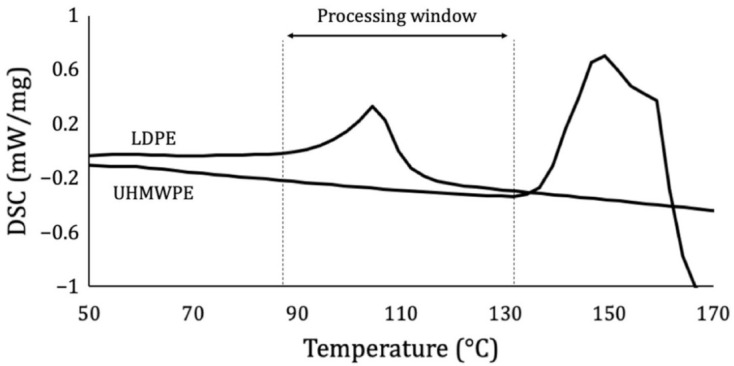
Definition of temperature window for processing through DSC analysis.

The preliminary observations demonstrated that a pressure of 1 bar was sufficient to ensure bonding, while values above 5 bar inferred excessive flattening of the bonded region (especially at high temperatures); therefore, joints made with pressures of 1, 2, 3, 4, and 5 bar were analyzed. Similar pressure ranges were adopted in previous studies [45,56,58,81] for thicker composites, making the adoption of this criterion for thin tapes a conservative premise to generate significant statistical data. As for the temperature, the DSC tests (Figure 2) demonstrated that the processing window ranged from approximately 90 °C (T_m_ of matrix) to 135 °C (T_m_ of reinforcement). Hence, samples prepared within this range [30] using temperatures of 90, 100, 110, 120, and 130 °C were studied. It was also noted that 1 min was enough to allow full bonding of the composites; given that potential industrial applications of the present study generally prefer fast and efficient processes, this value was adopted as the lowest time threshold. Times of 1, 2, 3, 4, and 5 min were considered. This exact time range and similar temperatures were also adopted in a previous study [56] involving PE, which demonstrated good results. After each bonding, samples were cooled to room temperature [62] via natural air convection to guarantee a low cooling rate and a consequent optimal composite performance [79].

Unlike Ye et al. [80], tabs in the terminations were not used to align the bonded samples in the load direction, because the tapes studied were flexible and so thin that this aspect would not influence the results, especially once the tabs used were made of paper and were approximately as thin as the tape itself.

Given the lack of a proper technical standard to test single-lap co-cured unidirectional UHMWPE/LDPE joints, the methods described in this work were carefully selected by analyzing previous studies and by adapting the existent ASTM standards. A test velocity of 1 mm/min was implemented to reproduce a quasi-static loading, as done in previous similar researches, that varied the velocity from 0.4 to 2 mm/min [45,51,56,57,58,63,80]. The shear resistance (τ) was calculated as the quotient between the maximum load (*F_max_*) and the bonded area [51,81], following Equation (1), where *L_J_* and *w* are the bonded length and tape width, respectively. Shear tests were carried out using an MTS 810 hydraulic universal testing machine with 5 MPa of grip pressure.

(1) $$\tau =\;F_{max}/(L_{J}w).$$

**Figure 3:**
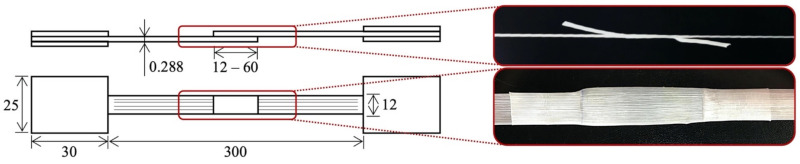
Single-lap co-cured sample configuration.

Specimens with 12, 24, 36, 48, and 60 mm of bonded length were tested (which translate into 1, 2, 3, 4, and 5 times the width of the tape) with the objective of enhancing the significance of data obtained in this study. Generally, although an increase in bonded area should cause an increase in maximum load, the maximum stress analyzed should ideally stay the same. The minimum bonded length assessed was equal to the width of the tape, as advised by the similar-purpose ASTM D5868 [62] and other authors [45,56]. The proportion of bonded length to distance between grips in the literature ranged from 16% to 25% [45,51,62]. The values adopted in this study, ranging from 4% to 20% in a constant 300 mm gage length, represent a conservative approach to maintain the bonded length far from the grips, thus making their influence neglectable. Therefore, as advised by the literature and manufacturer [23,75], one-layer samples with especially designed terminations were prepared for the joint shear tests, as shown in Figure 3. No strain gage was used due to the failure mechanics of the bonded composite, as further explained in the next section. The joints were examined via light microscopy (LM) with an Olympus (Tokyo, Japan) BXS1M microscope after the tests.

As expected, due to the high pressure and temperature, the reduction in viscosity of the matrix made the joints thinner than twice the thickness of the tape and wider than the original 12 mm. Since the stress calculation depends on the actual joint area, aiming to provide a more conclusive and precise analysis of the influence of processing parameters on the shear strength of the joint, the bonded overlapped areas were individually measured for each specimen by means of an appropriate software, and the thicknesses of the joints were measured using a digital caliper Mitutoyo (Kanagawa, Japan) 500-196-20B with 1 × 10^−2^ mm resolution at three distinct points to calculate the average and standard deviation values in each case.

### 2.3. Design of Experiment

Bearing in mind that, if the five values of each processing parameter (pressure, temperature, time, and joint length) were to form all possible combinations and then repeated at least three times each for the attainment of average and standard deviation values, it would involve at least 625 cure settings for a total of 1875 specimens and tests, which is clearly unfeasible. Thus, instead of adopting a traditional experimental routine, this study made use of the design of experiment (DoE) modeling technique through response surface methodology (RSM) to enable a comprehensive and yet feasible analysis. Statistically enhanced experimental models proposed by Montgomery [67], such as this one, were previously adopted in similar studies for the shear response of single-lap joints in composite materials [45,56].

RSM basically involves a set of statistical and mathematical techniques used for problem modeling in which dependent variables (i.e., response variables, e.g., force and stress) are influenced by controllable independent variables (i.e., input parameters, e.g., pressure, temperature, time, and joint length), where the objective is to optimize this response. The response surface is represented graphically through three-dimensional plots for each possible combination of two independent vs. one dependent variables, fulfilling the three available axes.

The first step of the RSM analysis is to define a regression model that adequately relates all independent variables with one dependent. The fit of this equation is improved if a second-order polynomial model is used. After the coefficients of the regression equation were estimated by the ordinary least squares method (OLS) [67], it is possible to understand the relationship between variables through three-dimensional surface plots and then represent them by two-dimensional contour plots (which are essentially projections of the surface plots), followed by optimizing the desired response variables as a function of the input parameters through a multiple response prediction [67]. In order to provide a more efficient estimation of the regression model coefficients, it is important to realize the experimental plan according to a second-order central composite design (CCD), where the quadratic interaction among the input variables (k) is defined by the axial points of a factorial design 2^k^ (in which the central point detects the lack of fit of the model), processing the independent variables to yield the interaction between them and the dependent variable.

Then, from the regression model calculated, it is possible to optimize the output variable to a minimum, target, or maximum value via an operational research routine that identifies the adjustment of a controllable factor to achieve the desired output value. The analysis was done using the Minitab V.19 software, which yielded the CCD-based experimental plan described in Table 2 involving 30 specimens.

## 3. Results and Discussion

### 3.1. Tensile Characterization of Matrix and Reinforcement

**Figure 4:**
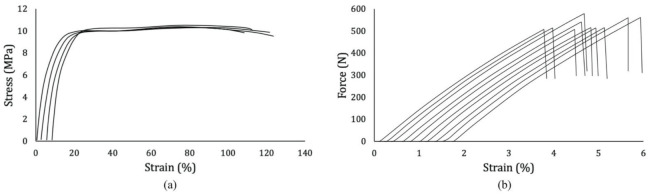
Tensile test results of (**a**) LDPE specimens 1–4 (left to right) and (**b**) UHMWPE yarn specimens 1–10 (left to right), plotted in offset.

The manufacturers supplied the density of both the fiber and the matrix as ρ_fiber_ = 975 kg/m^3^ and ρ_matrix_ = 913 kg/m^3^. The tensile behavior of both LDPE and UHMWPE yarns was demonstrated to be very consistent among samples, as shown by the minor standard deviations in Table 3 and Table 4, as well as the curves from Figure 4, which were plotted in an offset manner, because their similarity was so accentuated that the curves would have superimposed each other if plotted otherwise. For LDPE, the elastic modulus was calculated from 1% to 3% of strain, a region late enough to eliminate potential slack effects and early enough to make sure no plastic deformation took place. Given that it is not possible to measure with accuracy the transversal area of each tested yarn, the tensile resistance of UHMWPE was plotted in terms of force, which, through the fiber volume, can be converted into stress after the tensile analysis on the composite tape is done. The deformation of the yarns demonstrated a linear behavior until failure.

**Figure 5:**
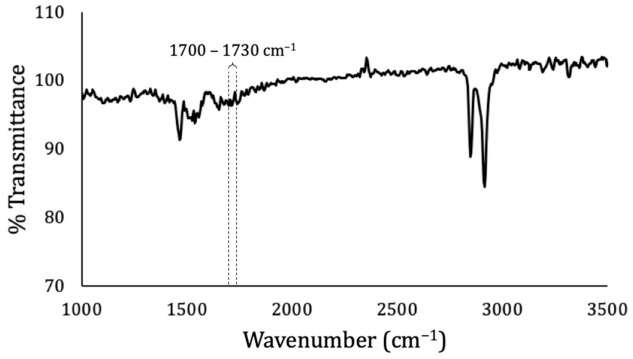
Fourier-transform infrared (FTIR) analysis of LDPE.

According to previous works and the “Precision and Bias” section of ASTM D638 [74,82], the tensile break stress of a virgin LDPE should be around 10.97 ± 0.36 MPa. The reprocessed LDPE studied herein had a tensile break stress of 10.63 ± 0.09 MPa. During reprocessing, the polymer went through thermal/oxidative and thermal/mechanical steps that could have degraded it depending on factors such as the catalyst used, processing conditions, and level of oxygen [9]. In the case of LDPE, the prevailing degradation mechanisms are chain branching and crosslinking, which can be noted by the presence of carboxylic groups in the FTIR spectrum of the material, identifiable from transmittance decays in a wavenumber range from 1700 to 1730 cm^−1^ [10,83]. The FTIR spectrum of LDPE studied herein (Figure 5) did not demonstrate a significant presence of carboxylic groups, whereas the elevated structural integrity of the material was concluded to have been due to its origin from a first or second reprocess or due to antioxidants being used during reprocessing.

The mechanical analysis conducted demonstrated that PE is a strong candidate for structural applications of reprocessed products, maintaining a strength equivalent to virgin PE, especially in the case of more branched polymers (such as LDPE) that are less sensitive to thermal degradation [7].

### 3.2. Tensile Characterization of the Composite

Five linear density measurements were carried out resulting in an average of 10,646.67 ± 122.66 dtex. Thus, according to the aforementioned fiber volume and the rule of mixture (ρ_fiber_ = 975 kg/m^3^ and ρ_matrix_ = 913 kg/m^3^), the expected thickness would be approximately 0.093 mm, agreeing well with the manufacturer data of 0.092 mm. Given the tape width of 12 mm, the transversal area could then be calculated as 1.116 mm^2^.

**Figure 6:**
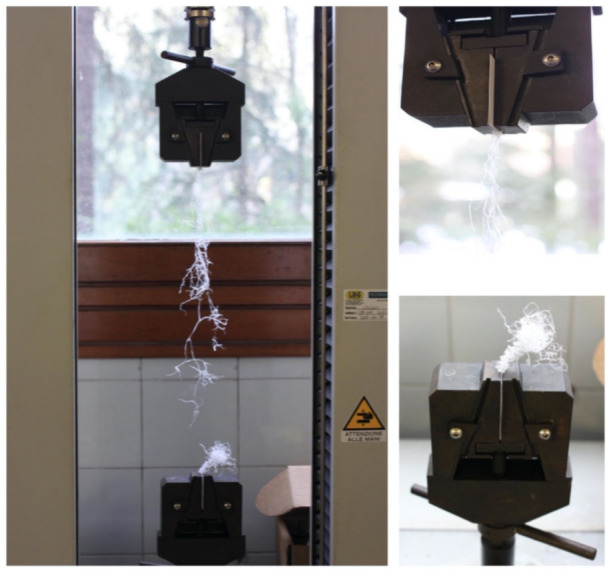
Typical tensile failure of the composite.

The fractures happened as expected: far from the grips and without material slippage (demonstrating the efficiency of the socket) (Figure 6). The failures were sudden, resulting in an immediate drop of the force to zero, indicating that all fibers from the tape failed approximately together, because the longitudinal axis of the tape was close enough to the load direction to leave the fibers were under the same stress.

**Figure 7:**
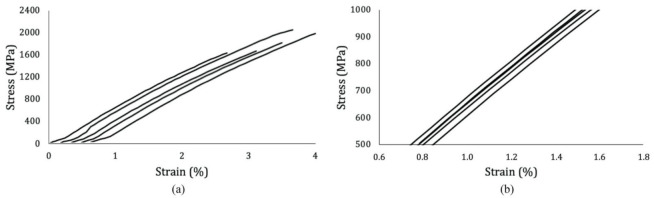
(**a**) Tensile test results of UHMWPE/LDPE tapes, specimens 1–5 (left to right) in offset; (**b**) post-slack zone considered for modulus calculation without offset.

Tensile failure force, stress, and strain are reported in Table 5. Results demonstrated the specimens to be 38% superior in terms of tensile strength and 16% in terms of modulus than recently studied UHMWPE/PE-wax SRCs [84]. The elastic modulus of each specimen was determined by eliminating the error inferred by the slack at the beginning of the tests, considering the region between 500 and 1000 MPa for modulus calculation. In this interval, the linear regression of all curves resulted in equations with over 99.9% of correlation. Thus, the angular coefficients of these equations, i.e., the slopes of the curves, were considered as the correct moduli. Figure 7 exhibits the stress–strain curves of the composites plotted in offset due to their almost identical shapes.

### 3.3. Micromechanical Analysis

It is a known fact that the properties of composites may vary depending on the literature source given the particular processing conditions that the matrix, fiber, and composite were subjected to in each singular study [85], whereby extra/interpolations of properties from state-of-the-art reviews are often the most reliable approach to estimate the mechanical behavior of composites. Furthermore, micromechanical-oriented studies on composite materials are rare in the literature, whereas most authors perform macromechanical tests and rely on the commercial properties of the constituents as provided by the manufacturers for a more thorough analysis. For this reason, taking advantage of the fact that the present work individually studies the components of the composite, an analytical determination of important properties is hereby provided. These include the area of the transversal section of the multifilament yarn, which is not possible to measure using regular metrology instruments beyond the costly procedure of measuring the diameter of a single fiber through SEM and multiplying the result by the number of fibers, as well as the yarn ultimate tensile stress and the yarn modulus. These properties are rarely available in the literature and could contribute to future studies, either for designing composites or for applications where there is no matrix (e.g., ropes for cargo-lifting and station-keeping of vessels). An isostrain condition and the rule of mixture approach were used for these calculations, due to the simplicity and yet extremely elevated accuracy of this method in this specific case of unidirectional thin composites [86].

With the values of ultimate tensile strength of the composite ($\sigma_{u,c}$) and the matrix ($\sigma_{u,m}$) of 1830 MPa and 10.6 MPa, respectively, found in the experiments above, and the fiber volume ($v_{f}$) of 65.6% provided by the composite manufacturer, it was then possible to calculate the ultimate tensile stress of the fibers ($\sigma_{u,f}$) according to Equation (2), resulting in 2785 MPa. The Hookean product of the maximum yarn tensile load of 540.8 N shown in Table 4 through the ultimate tensile stress of fibers yielded an individual transversal area of 0.194 mm^2^ for each yarn that was tested solely.

(2) $$\sigma_{u,f}=\;\left[\sigma_{u,c}-\sigma_{u,m}\left(1-v_{f}\right)\right]/v_{f}.$$

It is worth noting that the ultimate fiber breakage force in the composite, a product of $\sigma_{u,f}$ and 65.6% of the transversal area (i.e., 0.732 mm^2^), was 2038 N; this value can be considered equal to the ultimate tensile force of the composite itself of 2043 ± 203 N, demonstrating numerically that the composite fails as soon as fiber failure occurs.

(3) $$E_{1,f}=\;\left[E_{1,c}-E_{m}\left(1-v_{f}\right)\right]/v_{f}$$

The same rule of mixture approach could be used to calculate the longitudinal tensile modulus of the fiber ($E_{1,f}$) from the moduli of the composite ($E_{1,c}$) and matrix ($E_{m}$) found experimentally (Equation (3)), resulting in a value of 101 GPa.

### 3.4. Shear Characterization of Single-Lap Cured Joints

First, the cure of joints was carried out according to Table 2 in the specified order. Figure 2 shows that the temperatures selected were adequate, as they were all higher than the T_m_ of the matrix, allowing it to at least partially melt and bond the two adherends. Also, they were all lower than the T_m_ of the reinforcement, preserving its structural integrity. The triple-peak curve of UHMWPE after T_m_, as explained by Lacroix et al. [33], is due to its crystallinity and refers to the melting of a part of the orthorhombic phase, a lattice transition from orthorhombic to hexagonal, and a melting of the hexagonal phase, in that order.

**Figure 8:**
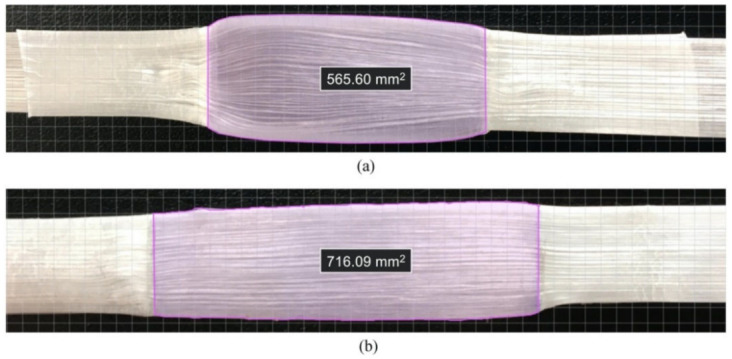
Joint area measurements of specimens (**a**) #26 and (**b**) #9 (not to scale).

After the cure, the total gage length of all specimens (*L_T_*) was measured to make sure no undesired longitudinal deformations occurred due to any potential shrinking of the cured regions. An average value of 299.6 ± 0.65 was found, concluding that any longitudinal deformation that might have taken place was negligible. Each individual joint thickness (*t_J_*) was measured in three different portions, with the aim of understanding how the processing parameters, mainly pressure and temperature, affect the out-of-plane compression of the joint (*Δt_J_*), which became thinner than two overlapped tapes after curing. The cured area was always higher than merely the product of the tape width and the designed length of the joint (*A_J_*), as the matrix flowed beyond the original 12 mm width when heat and pressure were applied. Since this area must be the one adopted for stress calculations to provide a precise analysis, each individual joint area after cure was measured (*A_J,real_*), and the consequent in-plane expansions (*ΔA_J_*) were determined. Examples of measurements of the actual joint area are displayed in Figure 8, which displays a more prominent flattening of specimen #26 that was cured at 3 bar, 110 °C, and 3 min than specimen #9 that was cured at 2 bar, 100 °C and 2 min. All of these dimensions are given in detail for all specimens in Table 6.

**Figure 9:**
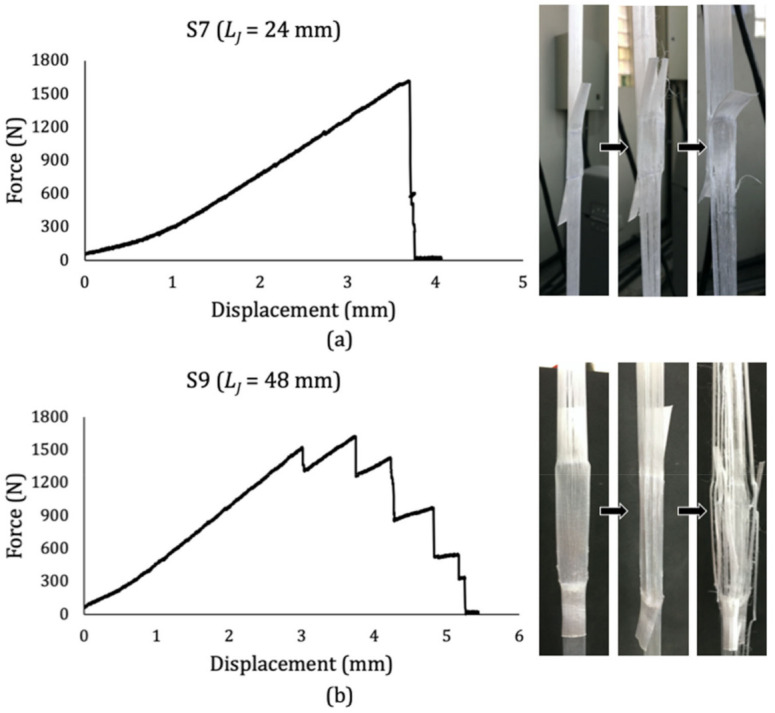
Typical failure progression of (**a**) short and (**b**) long co-cured joints.

Two main damage progression mechanisms were identified. For shorter joints (*L_J_* = 12 or 24 mm), linear loadings until failure were more common, where the joint remained mostly flat during the test while longitudinal cracks progressively appeared out of the joint. For longer joints (*L_J_* = 36, 48 and 60 mm), generally, the load rose to a point in which they became transversally wavy, which increased the local stress on the joint and allowed longitudinal cracks to take place within it. This effect could be explained by thinking of the specimens as a body constituted by three springs in series, where the portions out of the joint have a particular modulus defined by the tensile tests on the tape, and the joint in the middle has different properties, mainly due to twice the number of fibers in it. As the test goes on, the joint tends to deform less than the outer portions, and the stress concentration inferred to it causes this wavy-like deformation and consequent longitudinal cracks. Given the significantly weaker resistance of the material in the transversal axis, it leads to multiple subsequent longitudinal fractures along the length of the specimen, causing the load to drop several times before terminal failure. Figure 9 illustrates these mechanisms for specimen #7 (S7, Figure 9a) and specimen #9 (S9, Figure 9b), with *L_J_* = 24 mm and 48 mm, respectively.

Naturally, this phenomenon was more pronounced in specimens with longer joints due to their lower ability to longitudinally deform and their enhanced flattening during curing, where a higher amount of resin was displaced to the borders of the tape, leaving a larger central area with impaired transversal stiffness. No joint deflection was noted in the tests, probably because the specimens were quite thin.

**Figure 10:**
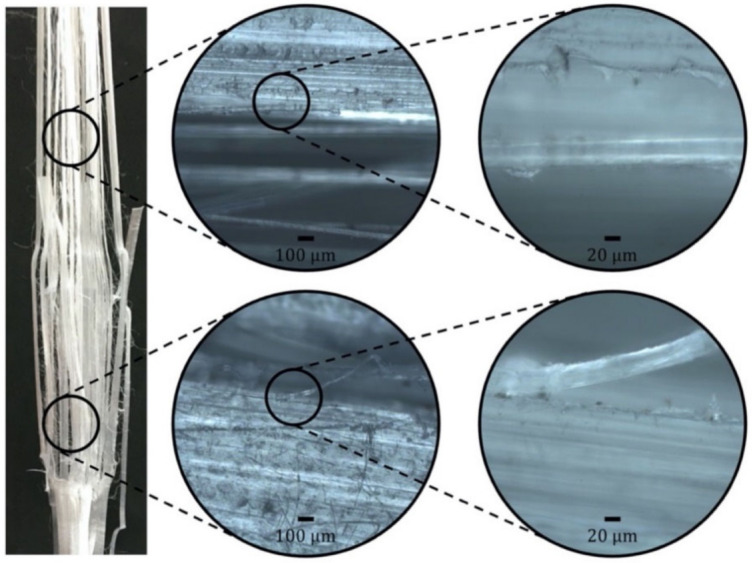
Typical debonding-induced longitudinal failure outside (**top**) and within (**bottom**) the joint.

With the aim of further investigating the fracture mechanics of this unidirectional composite, LM images revealed that the longitudinal cracks that preceded joint failure due to shear, which were more present in larger joint lengths due to the higher force levels and consequent enhanced transversal stress, were caused by debonding. As can be seen in Figure 10, this failure was typical of regions inside and outside the joint, meaning that processing was not a determinant factor. This finding makes sense, as, given the intrinsic low adhesion of UHMWPE, it is only logical that the stress level required to provoke debonding is smaller than that for a cohesive failure. This is understood as a positive feature of the composite, providing visually clear progressive failures before the terminal breakage of the joint rather than a catastrophic failure, which is an important advantage for structural composites by pointing out the eventual need for corrective maintenance.

From the experiment designed, the influence of processing parameters was calculated for average shear stress (τ) as an output variable. This assessment is important to fill the gap on co-cured thermoplastic tapes left by the single-lap shear technical standards, in an effort to identify the optimal processing conditions to generate a specimen that most accurately reproduces the behavior of the material when subjected to such loading and to study the fracture phenomena involved. Secondly, the optimization of shear is interesting for the actual performance of the joint (e.g., for industrial purposes), where load-bearing is the most practical and important feature of the material to be analyzed for potential applications.

Knowing that the maximum shear stress may coincide with the first load drop in the test (Figure 9a) or with a peak found several load drops after that (Figure 9b), for conservative purposes, the processing parameters optimization was made for the first failure stresses. This stress might be the actual breaking load or an early debonding-induced failure typical of joints with larger areas, from which the composite structural integrity is already compromised. Moreover, the optimization considering all maximum stresses provided the same optimal curing parameters and same correlation coefficient for the regression equation as that with the first failure stresses. The experimental stress and force results are shown in Table 7. These stresses were calculated considering the actual joint area (Table 6) to provide higher accuracy.

**Figure 11:**
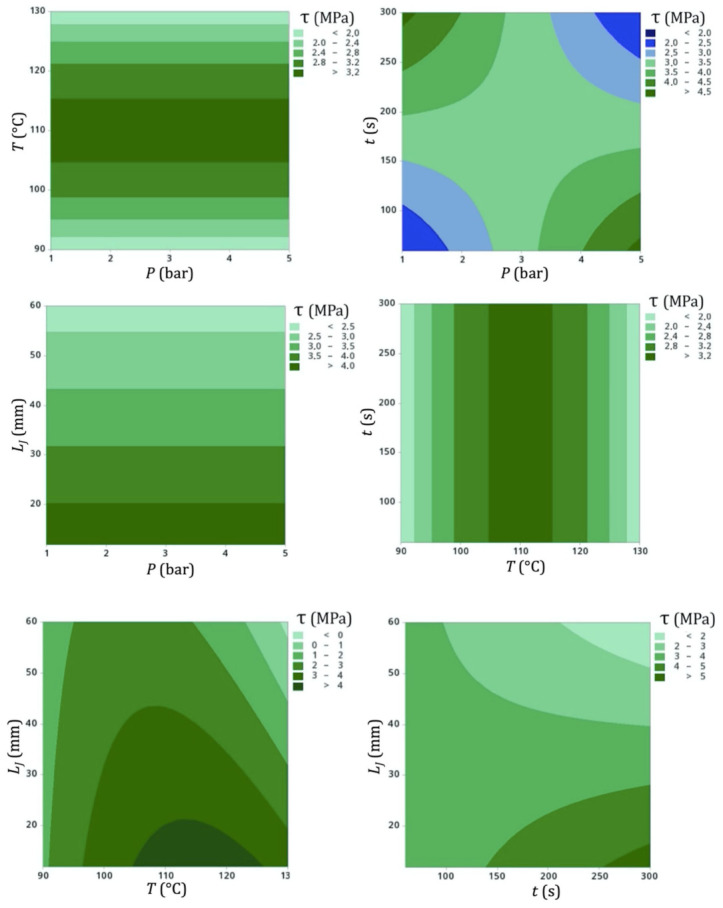
Contour plots of shear stress as a function of cure process parameters.

A first regression equation of shear was then determined using the OLS method by embracing all possible linear and quadratic combinations of the processing parameters in pairs, with a correlation coefficient of *R^2^* = 87.43%. However, it was possible to narrow down the influence of these parameters on shear stress by selecting only the interactions among them with *p*-values lower than 5%, namely, *L_J_*, *T^2^*, *P*·*t*, *T*·*L_J_*, and *t*·*L_J_*, thereby generating Equation (4), where τ is the shear stress (MPa), *P* is the pressure (bar), *T* is the temperature (°C), *t* is the time (s), and *L_J_* is the joint length (mm). Even though the new correlation coefficient became 81.99%, narrowing down the regression model to its significant terms is the advised approach to make the model simpler but reliable [67]. Figure 11 shows the contour plots of the model.

(4) $$\tau =3.3181-0.5242L_{J}-0.4142T^{2}-0.3363Pt-0.2213TL_{J}-0.2625tL_{J}$$

Upon analyzing the influence of processing parameters in pairs, it is interesting to highlight the major relevance of temperature with respect to other variables. When confronted with pressure and time, these parameters became practically indifferent for the outcome stress, an effect which was noticeably higher when the temperature was closer to 110 °C and lower when it drifted toward 90 °C or 130 °C. The key role of temperature in defining the final stress strength can be seen in Equation (4), where it is squared and linked to a relatively high 0.4 constant.

When pressure and time were compared, it was found that an average stress of 3.0 to 3.5 MPa could be achieved in most combinations, but it could be maximized if high pressures (4 to 5 bar) were applied for a time small enough to not allow the joint to suffer from exaggerated flattening (under 100 s) or if the cure adopted a low pressure (under 2 bar) for a time high enough for a successful bond between the adherend tapes.

The increase in joint length was linked to a decrease in shear stress in all related contour plots. Considering the Hookean perspective in Equation (1), this means that, although the force increased with higher joint areas (Table 7), this increase was not proportional. In other words, a constant increase in joint length led to a progressively smaller increase in force, resulting in decreasing stresses. This aspect can be explained by the fact that the stress concentration and structural imperfections inherent of the joint (such as the higher amount of material pushed outward the longitudinal centerline, with spread and less aligned fibers) became more prominent with larger joint areas, thus decreasing the stress despite increasing the force. The *L_J_* parameter takes into account all of these structural flaws and represents them quantitatively in the DoE. Hence, values of *L_J_* approximately equal or smaller than two times the adherend width were ideal for yielding higher shear stresses (which may be an interesting basis for the design of a proper technical standard test in the future). The aforementioned inversely proportional relationship for force and stress was also observed for joints on carbon-reinforced composites co-cured with steel [81].

**Figure 12:**
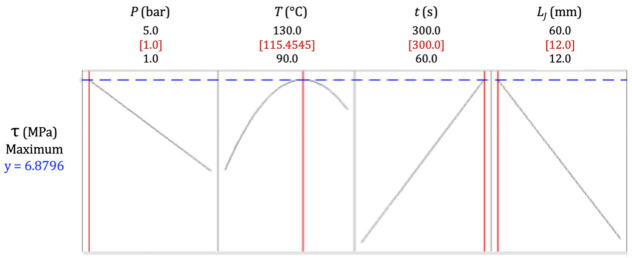
Optimized maximum shear stress and respective processing parameters obtained via multiple response prediction.

Taking the regression equation into account (Equation (4)), a multiple response prediction approach was used to optimize all combinations of input variables in order to estimate the maximum possible shear stress in an ideal case (represented by a composite desirability of 1.0) [67]. As shown in Figure 12, it is estimated that a shear force of 6.88 MPa could be achieved with a 12 mm long joint cured at 1 bar, 115.45 °C, and 300 s. More precisely, the optimal shear presented an averaged value of 6.88 ± 0.58 MPa within a fairly narrow 95% confidence interval between 5.68 and 8.08 MPa. This value is considered to be approximate to the ultimate tensile strength of the matrix alone (Table 3). Hence, knowing that the single-lap shear strength is mostly dependent on the matrix, this result can be seen as adequate.

Beyond the phenomenological aspect of this study, it is important to adopt these ultimate resistance values with caution when designing a structural application for the composite; not only may its resistance vary within the aforementioned statistical confidence interval, but applications involving constant loading may also infer an enhanced degradation to UHMWPE, which is known to degrade faster under creep.

The input values revealed by this optimization were very meaningful, evidencing that an ideal joint must be cured right after the endothermic transformation of LDPE, as seen in the DSC analysis (Figure 2), which took place between the T_m_ of the polymer and 115 °C, suggesting that the material was melted in its entirely. In other words, this temperature was the exact value to simultaneously provide the full melting of the matrix and cause less possible damage to the reinforcement, and it had to be applied for a time long enough to make sure the bond was well achieved, under a pressure sufficiently small to avoid excessive joint deformation and fiber misalignment.

An optimal lap joint processing at 115 °C was previously reported in the literature when co-curing PE with aluminum [56], where time was also reported to have a low influence on the resulting strength of the joint. A low effect of pressure in manufacturing thermoplastic UHMWPE-reinforced composites was reported by Hazzard et al. [87], who stated that higher pressures lead only to a small increase in shear strength and to a negligible effect on the laminate stiffness. It is important to underline that a low effect of pressure was present in the current study of thin PE SRCs, whereas it may be important when co-curing PP [30] or when joining PE to aluminum [56].

It is interesting to note that the shear resistance found both in the DoE optimization and in some of the unoptimized experiments carried out was superior even to the single-lap shear strength of UHMWPE/UHMWPE SRCs [88], which varied from 1.1 to 3.8 MPa depending on the processing parameters, demonstrating the extremely high performance of the reprocessed matrix SRC studied herein.

## 4. Conclusions

The present work analyzed the shear strength of single-lap co-cured self-reinforced polyethylene composite joints, by carrying out a thorough analysis on the processing conditions using the design of experiment approach of response surface. The experimental analysis allowed the authors to establish the following conclusions:

- A regression equation with a correlation coefficient of 82% was determined to estimate the in-plane shear resistance of joints as a function of their processing parameters. The most critical parameters and their combinations were *L_J_*, *T^2^*, *P*·*t*, *T*·*L_J_*, and *t*·*L_J_*, where *P* is the pressure, *T* is the temperature, *t* is the time, and *L_J_* is the joint length.
- Through the multiple response prediction method, it was possible to infer that a maximum joint shear strength of 6.88 MPa could be achieved if the joint was processed at 1 bar and 115 °C for 300 s.
- Temperature demonstrated to be the most influent parameter in determining the behavior of the joint, where the point at which the endothermic peak of the matrix ends was ideal (i.e., the temperature was high enough to fully melt the matrix but sufficiently low to not harm the reinforcement).
- The known low superficial adhesion of UHMWPE was responsible for the advantageous non-catastrophic debonding-induced progressive damage of the joint through longitudinal cracks at higher joint lengths.
- The tensile properties of the reprocessed matrix matched those of virgin LDPE, showing no presence of carboxylic groups in its structure and corroborating the literature on the ultimate recyclability of polyethylene.
- The properties of ultimate tensile stress and modulus of the reinforcing yarns were analytically assessed through the experiments on the composite. These properties are rarely seen in experimental studies given the difficulty of measuring the equivalent yarn diameter, and they can be quite useful when designing a composite or even for applications where there is no matrix (e.g., ropes for cargo-lifting and vessel station-keeping).

The authors encourage further research with this composite given its elevated mechanical properties, very low density, and unique recyclability among polymeric composites, thus showing its potential to fulfill existent and rising industrial needs, leading to a smarter use of materials and resources.

## Figures and Tables

**Table 1:** Properties of fiber (UHMWPE) and matrix (LDPE) obtained from [70,71,72,73,74].

UHMWPE	
Density (kg/m^3^)	975
Tensile strength (MPa)	3200–3400
Tensile modulus (MPa)	108,000–113,000
Elongation at break (%)	3.5
Compressive strength (MPa)	340
**LDPE**	
Density (kg/m^3^)	913
Tensile strength (MPa)	10
Tensile modulus (MPa)	83
Elongation at break (%)	129
Compressive yield strength (MPa)	5
Compressive modulus (MPa)	85

**Table 2:** Curing processing parameters for all specimens obtained through the response surface methodology (RSM) of design of experiment (DoE).

Specimen No.	1	2	3	4	5	6	7	8	9	10	11	12	13	14	15
*P* (bar)	2	4	2	4	2	4	2	4	2	4	2	4	2	4	2
*T* (°C)	100	100	120	120	100	100	120	120	100	100	120	120	100	100	120
*t* (min)	2	2	2	2	4	4	4	4	2	2	2	2	4	4	4
*L_J_* (mm)	24	24	24	24	24	24	24	24	48	48	48	48	48	48	48
**Specimen No.**	**16**	**17**	**18**	**19**	**20**	**21**	**22**	**23**	**24**	**25**	**26**	**27**	**28**	**29**	**30**
*P* (bar)	4	3	3	3	3	1	5	3	3	3	3	3	3	3	3
*T* (°C)	120	110	110	110	110	110	110	90	130	110	110	110	110	110	110
*t* (min)	4	3	3	3	3	3	3	3	3	1	5	3	3	3	3
*L_J_* (mm)	48	36	36	36	36	36	36	36	36	36	36	12	60	36	36

**Table 3:** Tensile results of LDPE.

LDPE						
Specimen No.	1	2	3	4	x̄	Σ
σ_break_ (MPa)	10.6	10.6	10.7	10.7	10.6	0.1
ε_break_ (%)	124	106	118	104	113	9
E (MPa)	135	114	126	132	127	9

**Table 4:** Tensile results of UHMWPE.

UHMWPE												
Specimen No.	1	2	3	4	5	6	7	8	9	10	x̄	Σ
F_break_ (N)	517	525	587	550	516	525	525	524	567	568	540	25
ε_break_ (%)	3.75	3.75	4.25	4.00	3.66	3.83	3.75	3.75	4.08	4.17	3.90	0.21

**Table 5:** Tensile results of the composite.

UHMWPE/LDPE							
Specimen No.	1	2	3	4	5	x̄	Σ
F_break_ (N)	1824	2293	1868	2026	2202	2043	203
σ_break_ (MPa)	1635	2055	1674	1815	1973	1830	182
ε_break_ (%)	2.67	3.51	2.80	3.04	3.39	3.08	0.36
E (GPa)	66.5	66.9	65.6	65.8	66.4	66.2	0.5

**Table 6:** Dimensions of the single-lapped specimens after curing.

Specimen No.	*L_T_* (mm)	*t_J_* (mm)	*Δt_J_* (%)	*A_J_* (mm^2^)	*A_J,real_* (mm^2^)	*ΔA_J_* (%)
1	299.5	0.58 ± 0.01	0.69	288	387.24	34.46
2	300.6	0.57 ± 0.03	1.62	288	409.97	42.35
3	299.3	0.49 ± 0.03	14.35	288	394.54	36.99
4	300.0	0.39 ± 0.04	31.71	288	375.13	30.25
5	299.4	0.54 ± 0.02	5.67	288	376.54	30.74
6	299.9	0.54 ± 0.01	6.25	288	400.91	39.20
7	299.1	0.51 ± 0.04	11.46	288	388.81	35.00
8	300.1	0.43 ± 0.02	25.93	288	400.58	39.09
9	299.8	0.43 ± 0.02	24.77	576	716.09	24.32
10	300.5	0.46 ± 0.02	19.56	576	754.37	30.97
11	299.1	0.44 ± 0.02	24.19	576	850.49	47.65
12	299.2	0.39 ± 0.04	31.71	576	804.66	39.70
13	300.8	0.47 ± 0.01	18.40	576	711.38	23.50
14	299.2	0.47 ± 0.03	18.40	576	844.98	46.70
15	298.9	0.51 ± 0.04	11.46	576	838.14	45.51
16	297.9	0.42 ± 0.03	26.50	576	831.01	44.27
17	300.0	0.45 ± 0.02	21.88	432	593.68	37.43
18	300.2	0.47 ± 0.02	18.98	432	552.15	27.81
19	299.5	0.48 ± 0.01	17.25	432	582.60	34.86
20	300.0	0.41 ± 0.01	28.24	432	657.73	52.25
21	300.2	0.44 ± 0.03	23.61	432	608.05	40.75
22	299.8	0.44 ± 0.02	24.19	432	601.55	39.25
23	300.0	0.57 ± 0.04	0.46	432	496.27	14.88
24	298.5	0.50 ± 0.03	13.77	432	683.68	58.26
25	299.5	0.46 ± 0.04	20.72	432	555.63	28.62
26	300.6	0.38 ± 0.02	33.45	432	565.60	30.93
27	299.9	0.42 ± 0.04	27.08	144	209.80	45.69
28	299.0	0.36 ± 0.04	36.92	720	1014.54	40.91
29	299.2	0.45 ± 0.03	22.45	432	589.90	36.55
30	299.3	0.53 ± 0.01	8.56	432	581.56	34.62

**Table 7:** First failure shear force (F) and shear stress (τ).

Specimen No.	1	2	3	4	5	6	7	8	9	10	11	12	13	14	15
F (kN)	1.18	1.39	0.96	1.61	1.42	1.36	1.62	1.38	1.53	2.33	2.11	1.26	2.09	1.86	1.19
τ (MPa)	3.05	3.39	2.42	4.28	3.78	3.39	4.17	3.44	2.13	3.09	2.48	2.52	2.94	2.20	1.42
**Specimen No.**	**16**	**17**	**18**	**19**	**20**	**21**	**22**	**23**	**24**	**25**	**26**	**27**	**28**	**29**	**30**
F (kN)	0.91	1.92	1.76	1.47	1.94	2.16	1.84	0.91	1.05	1.75	1.92	0.91	3.10	2.07	1.76
τ (MPa)	1.10	3.24	3.20	3.37	2.94	3.55	3.07	1.83	1.54	3.15	3.40	4.33	3.06	3.50	3.03

## Data Availability

The raw/processed data required to reproduce these findings cannot be shared at this time as the data also form part of an ongoing study.
